# Treatment strategy to maximize the treatment outcome of spinal dural arteriovenous fistula after initial endovascular embolization attempt at diagnostic angiography

**DOI:** 10.1038/s41598-021-89407-w

**Published:** 2021-05-11

**Authors:** Heui Seung Lee, Hyun-Seung Kang, Sung Min Kim, Chi Heon Kim, Seung Heon Yang, Moon Hee Han, Chun Kee Chung

**Affiliations:** 1 Department of Neurosurgery, Hallym University Sacred Heart Hospital, College of Medicine, Hallym University, Anyang, Korea; 2 Department of Neurosurgery, Seoul National University College of Medicine, 101 Daehak-ro, Jongno-gu, Seoul, 110-744 Korea; 3 Department of Neurology, Seoul National University College of Medicine, Seoul, Korea; 4 Department of Radiology and Neurosurgery, Veterans Health Service Medical Center, Seoul, Korea

**Keywords:** Neurology, Neurological disorders, Neuro-vascular interactions

## Abstract

Initial attempt of endovascular treatment (EVT) for spinal dural arteriovenous fistula (SDAVF) is preferred because of concurrent diagnosis and treatment. However, outcomes following further treatment with initial EVT are not well studied. We retrospectively reviewed 71 patients with SDAVF to evaluate treatment outcomes of SDAVF after an initial EVT attempt. Pretreatment and posttreatment functional states were assessed by the Aminoff-Logue scale (ALS). In the case of incomplete occlusion or recurrence, overall outcomes after further treatments were compared. Of the 71 patients, 56 underwent initial EVT. Complete occlusion was achieved by initial EVT in 37 of 56 patients (66.1%). Multiple feeders were more frequently observed in patients with incomplete occlusion than complete occlusion after initial EVT (73.7% vs. 27%, P < 0.001). Among 19 patients with incomplete occlusion upon initial EVT, 14 underwent additional surgery, 13 of whom (92.9%) obtained improved or stationary functional outcomes. Functional improvement was not observed in patients who had repeated EVT or follow-up without further treatment. Recurrence was observed in 8 of 37 patients with complete occlusion upon initial EVT. Additional surgery achieved improved functional outcomes in cases of incomplete occlusion of SDAVF after the initial EVT attempt or recurrence rather than repeated EVT or follow-up.

## Introduction

Although the surgical outcome of spinal dural arteriovenous fistula (SDAVF) is excellent, with a success rate over 95%^1–6^, initial attempt of endovascular treatment (EVT) at the same session of diagnostic spinal angiography for spinal dural arteriovenous fistula (SDAVF) has become a preferred approach because of the benefit from concurrent diagnosis and treatment with a 70–90% rate of complete obliteration. However, overall outcomes following further treatment with initial EVT are not well studied^4,7–12^.

Although a comparison of treatment outcomes between EVT alone and surgery has been performed^4^, there is no consensus for further treatment when SDAVF is incompletely occluded or recurs after initial EVT.

To find the optimal individual treatment strategy for incomplete occlusion or recurrence by initial EVT, we reviewed all SDAVF patients treated in our hospital under the policy of an initial EVT attempt and elucidated factors associated with incomplete occlusion by initial EVT. Additionally, we compared overall outcomes after further treatment in cases of incomplete occlusion or recurrence according to different treatment options after initial EVT.

## Methods

### Patient data collection and preoperative evaluation

**Figure 1:**
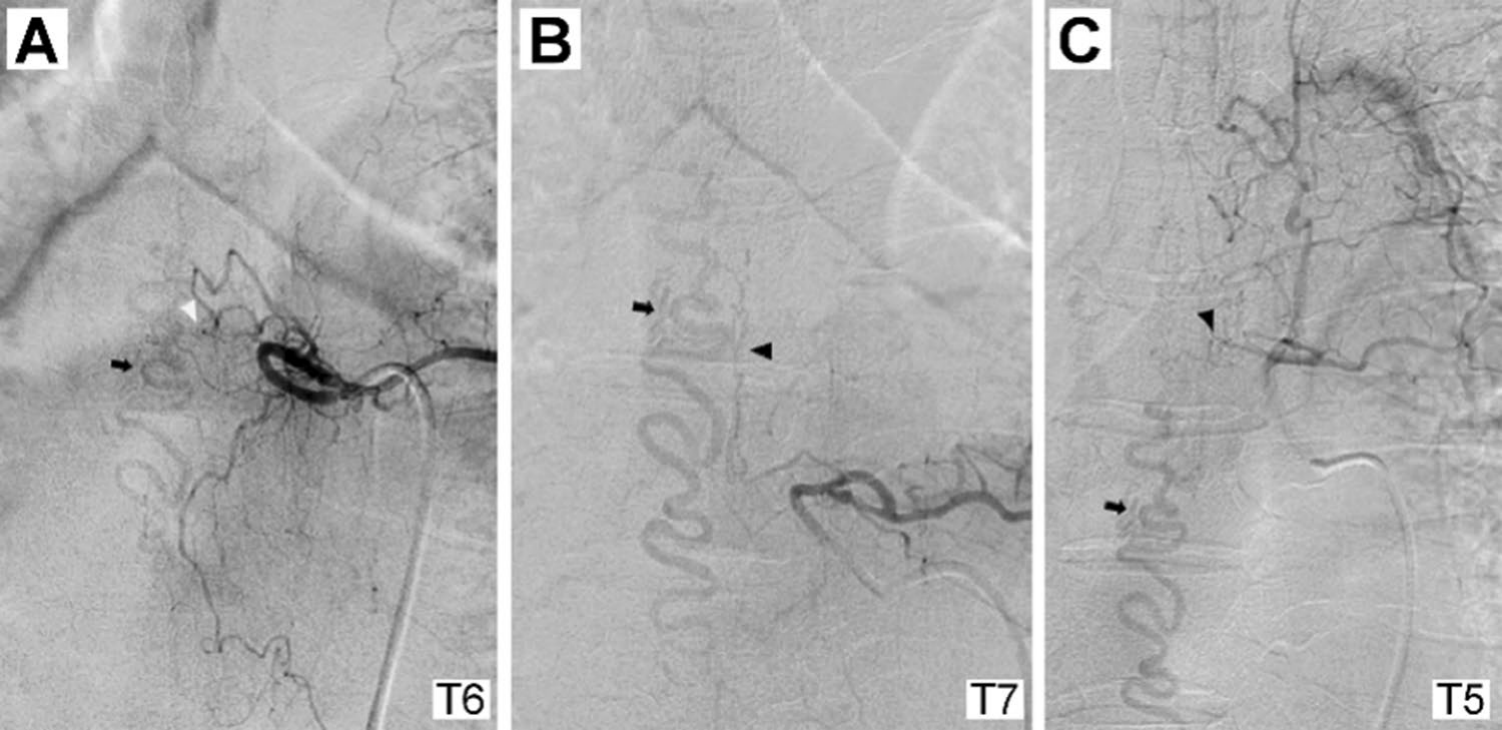
A case of thoracic dural arteriovenous fistula at the T6 level with multiple feeders was defined in the present study. (**A**) Angiogram of the left T6 segmental artery demonstrating the main arterial feeder of the radiculomeningeal artery (white arrowhead) arising from the left T6 segmental artery connected to the engorged perimedullary vein (arrow). (**B**) and (**C**) show engorgement of the perimedullary vein (arrow) after visualization of the dural arteriovenous fistula by collateral channels (black arrowheads) on the angiograms of the left T7 and T5 segmental arteries.

We retrospectively reviewed all patients newly diagnosed at our institute with spinal arteriovenous malformation that was confirmed by spinal angiography between January 2004 and March 2019. Among those with spinal arteriovenous malformations, adult patients (age ≥ 18 years) who had angiographical findings compatible with type I SDAVF according to the classification by Takai et al. were selected. The patients’ medical records were reviewed to extract demographic information, functional status before treatment and initial treatment after diagnostic spinal angiography. Multiple feeders were defined when arteriovenous shunt flow of more than two different levels was visualized on the spinal angiogram (Fig. 1).

The preoperative functional status of patients was assessed by the modified Aminoff-Logue scale (ALS) (Table 1)^13^. ALS scores of 5 or more were categorized as functionally severe cases^10,14^. Patients with mixed intracranial and spinal dural fistulas, SDAVF with comorbid structural malformations, such as spinal dysraphism, or spine or spinal cord tumors were excluded.

**Table 1:** The modified Aminoff-Logue scale for the assessment of patients’ functional state.

Grade	Definition
**Gait**	
1	Leg weakness, abnormal gait or stance, but no restriction of activity
2	Restricted activity
3	Requiring 1 stick for walking
4	Requiring 2 sticks, crutches, or walker
5	Confined to wheelchair
**Micturition**	
1	Hesitancy, frequency, urgency
2	Occasional urinary incontinence or retention
3	Total urinary incontinence or retention

### Treatments

The treatment policy of our hospital for patients diagnosed with SDAVF by spinal angiography is as follows. EVT is initially attempted for the first treatment to obliterate SDAVF if feasible. Initial surgical treatment without EVT is decided when EVT is considered not feasible based on spinal angiography in the following situations: (1) when the origin of either the anterior spinal artery (ASA) or posterior spinal artery (PSA) shares arterial feeders with the SDAVF or (2) when feeder selection by endovascular microcatheter is not feasible due to the small size or tortuosity of the arterial feeder.

Treatment-related complications were identified from medical records as well as relevant postoperative spine MRI scans.

### Endovascular treatment

*N*-Butyl-2-cyanoacrylate (NBCA) was used as a liquid embolic material in the present study^7^. Complete embolization was defined as the liquid embolic material filling the proximal portion of the draining veins after filling the fistulous channel^15,16^.

### Surgical obliteration of SDAVF

Surgical treatment of SDAVF consisted of laminotomy targeting the level of the AVF without violating the facet joint, opening the dura, and disconnecting the draining vein via coagulation and resection of the vessel. Temporary clipping of the drainage vein was used to confirm disappearance of the AV shunt flow, which was identified by either Doppler sonography or intraoperative ICG angiogram.

### Posttreatment follow-up and assessment of treatment outcomes

In the case of complete occlusion by either initial EVT or surgery, patient evaluation and follow-up spine MRI were performed 6 months after initial treatment. When complete occlusion was not achieved by the initial treatment, follow-up spinal angiography was performed at post-treatment one month to determine the additional treatment. In patients who underwent additional treatment because of incomplete occlusion after initial EVT, reevaluation with spine MRI was scheduled between 10 and 12 months after the initial EVT. For patients who underwent surgery due to infeasible EVT, spinal angiography was performed immediately after the surgery to confirm occlusion of the AV shunt. Angiographic results were defined as follows: complete obliteration (complete embolization or surgical interruption of all fistulas or total resection of the whole nidus) and partial obliteration (partial embolization or surgical interruption of the fistula or partial resection of the nidus)^16^.

Recurrence of SDAVF during follow-up was defined as a newly increased perimedullary flow void with contrast enhancement of perimedullary vessels on spine MRI with gadolinium (Gd) enhancement or visualization of the AV fistula on spinal angiogram at the same level as the AV shunt that was previously obliterated^17^. Follow-up spinal angiography was performed as follows: (1) in cases of incomplete obliteration by the initial EVT, at post-treatment 1 month; (2) in cases of complete occlusion by the initial treatment, when patients showed neurological deterioration and findings of SDAVF recurred or myelopathy progression on follow-up spine MRI.

Patient functional outcomes were assessed by the difference between ALS score at the initial treatment and that recorded at the last clinical follow-up. Outcomes were defined as follows: ‘improved’ when there was a decrease in the ALS score by 1 or more, ‘stationary’ when there was no difference, and ‘worsened’ when there was an increase in the ALS score by 1 or more.

### Statistical analysis

Statistical analyses were performed with the computing environment R Version 3.6.3 (R Development Core Team, 2017. R: A language and environment for statistical computing. R Foundation for Statistical Computing, Vienna, Austria. URL https://www.R-project.org/). Normality of the data was tested using the Shapiro–Wilk test. The Mann–Whitney U test was applied to evaluate statistically significant differences between the mean values of continuous variables. The correlation of pretreatment ALS and ALS differences between pretreatment and posttreatment was assessed using Spearman’s rank correlation. Fisher’s exact test or the chi-square test was performed to determine significant differences between categorical variables; P values less than 0.05 were considered statistically significant.

### Ethics approval

This study was approved by the Seoul National University College of Medicine/Seoul National University Hospital Institutional Review Board (IRB No. H-1910-158-1073) and the present study was performed in accordance with the Declaration of Helsinki. All study participants provided written informed consent.

## Results

### Patient characteristics and angiographic results after initial treatment

Among 121 patients with spinal AV malformations, 71 (58.7%) (60 males/11 females, mean age 59.5 ± 11.8 years) had angiographic findings compatible with type I spinal SDAVF and were included in the present study. The baseline characteristics of the patients are shown in Table 2. The mean follow-up duration was 36 ± 35 months (range 6–160 months).

**Table 2:** Clinical information and radiological findings for 71 patients diagnosed with spinal dural arteriovenous fistula.

	SDAVF	Initial EVT
	N = 71	N = 56
Age (mean ± SD, years)	59.5 ± 11.8	58.8 ± 11.9
Sex M/F (male %)	60/11 (84.5%)	46/10 (82.1%)
Hypertension (%)	21 (29.6)	13 (23.2)
**Initial presentation**		
Back pain	9 (12.7)	7 (12.5)
Paresthesia	55 (77.5)	41 (73.2)
Lower extremity weakness	52 (73.2)	43 (76.8)
Voiding difficulty	36 (50.7)	29 (51.8)
Constipation	19 (26.8)	16 (28.6)
Subarachnoid hemorrhage	4 cases of cervical SDAVF	4 (7.1)
Pretreatment symptom duration^†^ (mean ± SD, months)	9.8 ± 8.1 [range 0–36 months; Median, 7 months; IQR, 4, 12.5 months]	9.89 ± 7.95 [range 0–30 months; Median, 7 months; IQR, 4, 14 months]
Severe cases (ALS ≥ 5)	15 (21.1)	12 (21.4)
**Locations**		
C	11 (15.5)	9 (16.1)
T	45 (63.4)	34 (60.7)
L	12 (16.9)	10 (17.9)
	3 (4.2)	3 (5.4)
Pretreatment myelopathy	61 (87.3)	49 (87.5)
Multiple feeders (≥ 2)	32 (45.1)	24 (42.9)
Follow-up duration^‡^ (mean ± SD, months)	36 ± 35 [range 6–160 months; Median 24 months; IQR: 10–49 months]	35 ± 34 [range 6–160 months; Median 24 months; IQR: 10.5–42 months]

*SDAVF* spinal dural arteriovenous fistula; *ALS* Aminoff-Logue scale; *C* cervical; *T* thoracic; *L* lumbar; *S* sacral; *MRI* magnetic resonance image; *SI* signal intensity; *VB* vertebral body.

^†^^, ‡^Duration is reported as both the mean ± standard deviation (SD) and median with interquartile range (IQR).

Initial EVT was feasible in 56 patients. However, the initial attempt of EVT was deemed infeasible in 15 patients for the following reasons: 6 had ASA or PSA originating from the SDAVF feeder, and 9 had arterial feeders that were too small for EVT. These 15 patients underwent surgery instead of EVT for the initial treatment, and complete occlusion was achieved in all cases postoperatively.

Complete occlusion of the SDAVF was achieved in 37 of 56 patients (66.1%) with initial EVT. Patients with multiple feeders were more likely to have incomplete occlusion of the SDAVF after the initial EVT (P < 0.001). Additionally, patients with cervical SDAVFs had a lower likelihood of complete occlusion than did those with SDAVFs at other locations by initial EVT (3/9, 33.3% vs. 34/47, 72.3%, respectively) (P = 0.04) (Table 3).

**Table 3:** Angiographic results after the initial endovascular treatment of 56 patients with spinal dural arteriovenous fistula.

Initial EVT	Complete occlusion	Incomplete occlusion	P value
(N = 56)	37 (66.1)	19 (33.9)	
Multiple feeder (%)	10 (27)	14 (73.7)	< 0.001
**Location (% per location) [number of cases with multiple feeders]**			
C	3 (33.3) [1, 33.3%]	6 (66.7) [6, 100%]	0.04
T	22 (64.7) [7, 31.8%)	12 (35.3) [7, 58.3%]	
L	9 (90) [2, 22.2%]	1 (10) [1, 100%]	
S	3 (100) [0]	0	

Values represent numbers of patients (%).

*C* cervical; *T* thoracic; *L* lumbar; *S* sacral.

**Figure 2:**
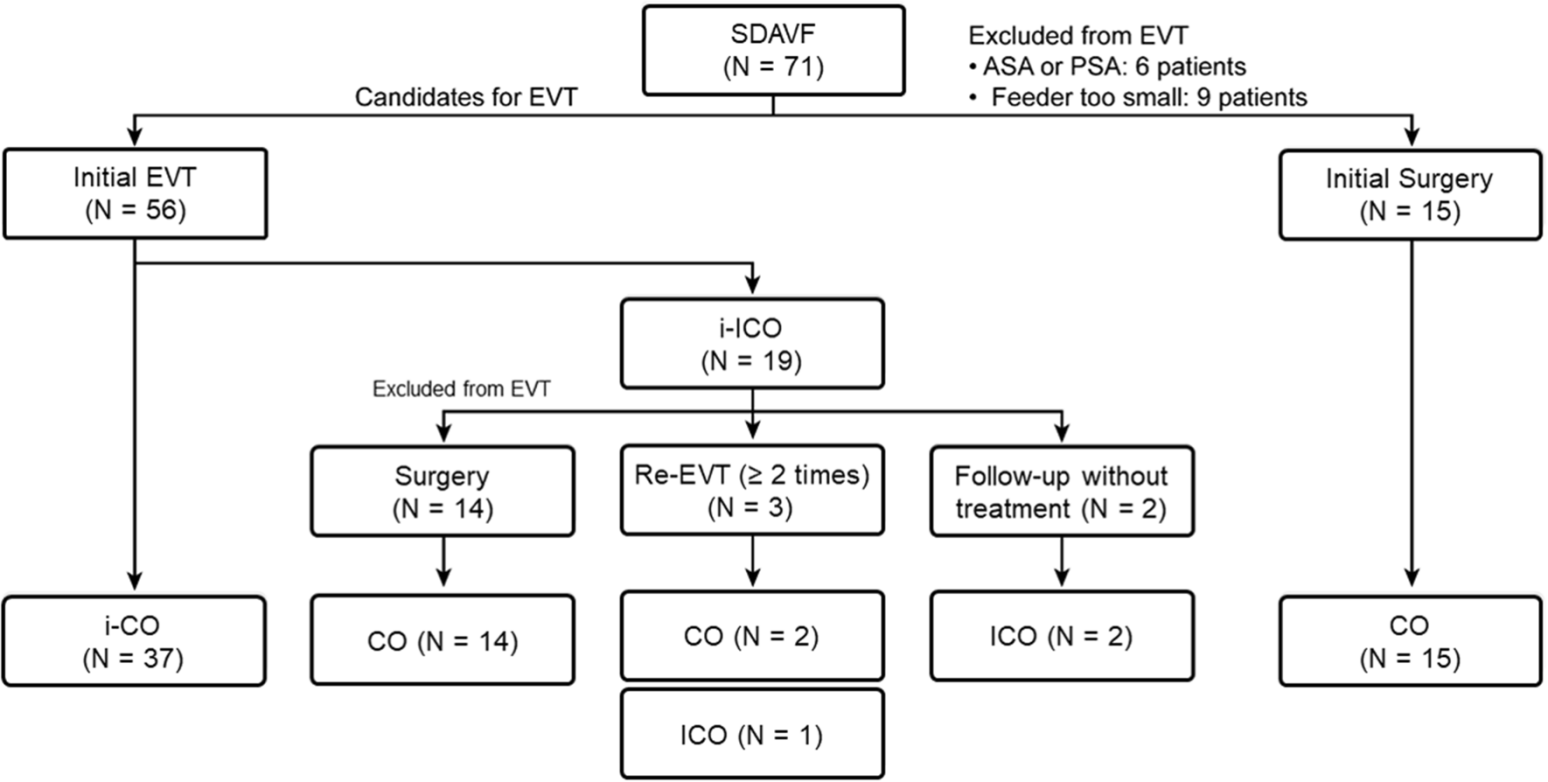
Flow chart of treatment decisions upon initial diagnosis of SDAVF and during follow-up periods and angiographic results after each treatment. *SDAVF* spinal dural arteriovenous fistula; *ASA* anterior spinal artery; *PSA* posterior spinal artery; *EVT* endovascular treatment.

Incomplete occlusion occurred in 19 patients after initial EVT. Additional surgery achieved complete occlusion of the SDAVF in 14 patients, whereas repeated EVT achieved complete occlusion of the SDAVF in 2 of 3 patients (Fig. 2). The mean time to additional treatment after incomplete occlusion via initial EVT was 58 ± 29 days (range 17–97 days). There were no instances of spontaneous occlusion of the SDAVF in the 2 patients who were only observed and followed up after incomplete occlusion via initial EVT.

Treatment-related complications occurred in 4 patients with cervical SDAVF who underwent initial EVT: lateral spinal cord infarction at the C1 level (1), posterior inferior cerebellar artery (PICA) infarction (2), and vertebral artery dissection (1).

### Treatment outcomes

Of the 71 patients with SDAVF, 57 (80.3%) exhibited stationary or improved functional outcomes (36 (50.7%) with improved outcomes and 21 (29.6%) with stationary outcomes)). However, 14 patients (19.7%) had worsened functional outcomes at the last follow-up (Table 4). There was no significant difference in treatment outcomes according to the location of the SDAVF.

**Table 4:** Functional outcomes of 71 patients with spinal dural arteriovenous fistula at last follow-up.

	Improved N = 36	Stationary N = 21	Worsened N = 14	P value
**Locations (% per location)**				0.1
C	2 (18.2)	6 (54.5)	3 (27.3)	
T	28 (62.2)	9 (20)	8 (17.8)	
L	6 (50)	4 (33.3)	2 (16.7)	
S		2 (66.7)	1 (33.3)	
**Pretreatment severity (% per group)**				0.13
ALS 0–4	25 (44.6)	18 (32.1)	13 (23.2)	
ALS 5–8	11 (73.3)	3 (20)	1 (6.7)	
Pretreatment symptom duration (mean ± SD, months)	11.4 ± 9.2	7.1 ± 6.6	8.14 ± 6.4	0.21
**Treatment options**				
CO by iSurgery (N = 15)	10 (C, 1; T, 8; L, 1)	4 (C,1; T, 2; L,1)	1 (T, 1)	
CO by iEVT (N = 37)	18 (T, 13; L, 5)	9 (C, 1; T, 4; L, 2; S, 2)	10 (C, 2; T, 5; L, 2; S1)	
ICO by iEVT + Surgery (N = 14)	8 (C, 1; T, 7)	5 (C, 3; T, 2)	1 (T, 1)	
ICO by iEVT + Re-EVT (N = 3)	0	2 (C, 1; L, 1)	1 (C, 1)	
ICO by iEVT + FU (N = 2)	0	1 (T, 1)	1 (T, 1)	
**Complications**				
SDAVF Location/complication (Number of patients)		C1/spinal cord infarction (1)	C1-2/PICA infarction (2)	
			C1-2/VA dissection (1)	

Values represent numbers of patients (%).

*ALS* Aminoff-Logue scale; *CO* complete occlusion; *iEVT* initial EVT; *iSurgery* initial Surgery; *ICO* incomplete occlusion; *Re-EVT* repeated EVT; *FU* follow-up.

**Figure 3:**
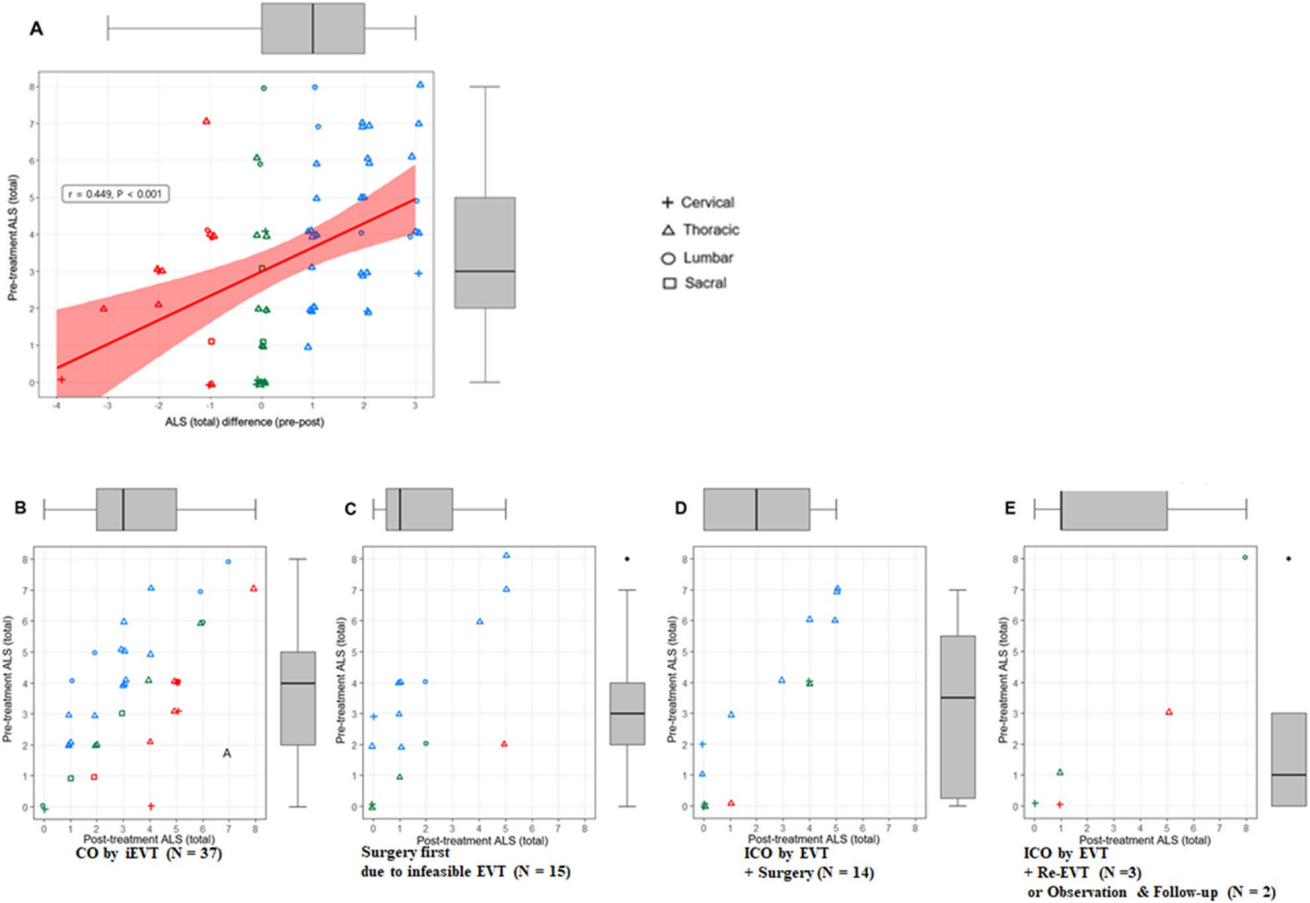
Comparison and distribution of pretreatment and posttreatment ALS scores of 71 patients with spinal dural arteriovenous fistula. (**A**) Scatter plots of the pretreatment ALS (total) versus ALS difference between pre- and post-treatment in 71 patients. r represents Spearman's rank correlation coefficient. (**B**–**E**) Scatter plots of pre- versus post-treatment ALS (total) values among patients who underwent different treatment courses. Blue dots denote improved functional outcomes; green dots denote stationary functional outcomes; red dots denote worsened outcomes. *ALS* Aminoff-Logue scale; *SDAVF* spinal dural arteriovenous fistula; *EVT* endovascular treatment; *ALS* Aminoff-Logue scale; *CO* complete occlusion; *iEVT* initial EVT; *ICO* incomplete occlusion; *Re-EVT* repeated EVT; *FU* follow-up.

Although there was no statistical significance, the proportion of patients with improved functional outcome after treatment was more frequently reported in the group with pretreatment ALS scores of 5–8 (more severe, n = 15) than in the group with pretreatment ALS scores of 0–4 (less severe, n = 56) (11/15, 73.3% vs. 25/56, 44.6%, respectively; P = 0.13). Worsened outcomes were reported in 13 patients (23.2%) among 56 with pretreatment ALS scores of 0–4, whereas one of 15 patients (6.7%) with pretreatment ALS scores of 5–8 had worsened outcomes. The distribution of the difference in ALS score between pre- and post-treatment (ALS difference (pre-post)) is shown in Fig. 3A, and the pretreatment ALS score had a positive correlation with the ALS difference (pre-post) (r = 0.449, P < 0.001).

The distribution of the patients’ pre- and post-treatment ALS scores is shown in Figure 3B–E. There was no significant difference in pretreatment symptom duration between the groups according to functional outcomes at the last follow-up (P = 0.21) (Table 4).

**Figure 4:**
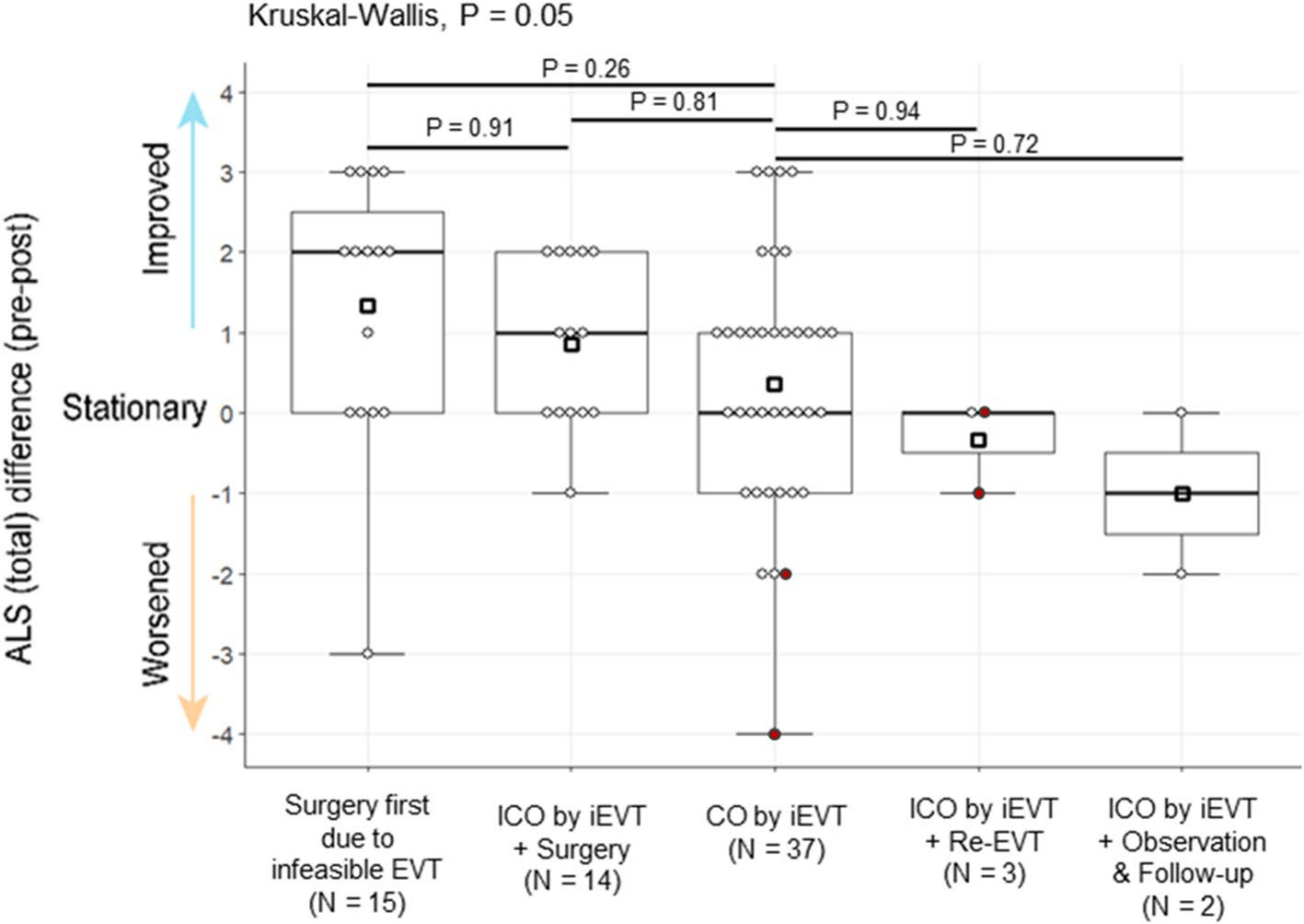
Scatter bar plot showing the comparison of functional outcomes of patients who underwent different treatment courses. Circles denote each patient; red-filled circles denote patients with complications; squares denote the mean value of ALS (total) difference (pre-post). *EVT* endovascular treatment; *ALS* Aminoff-Logue scale; *CO* complete occlusion; *iEVT* initial EVT; *ICO* incomplete occlusion; *Re-EVT* repeated EVT; *FU* follow-up.

Of the 19 patients with incomplete occlusion by initial EVT, 14 underwent additional surgery to achieve complete occlusion of the SDAVF, and 13 of these patients (92.9%) showed improved or stationary functional outcomes (8 improved; 5 were stationary; 1 worsened) (Table 4 and Fig. 4). Among patients with incomplete occlusion by initial EVT, salvage treatment by additional surgery resulted in the treatment outcome which is equivalent to the group who underwent surgery without initial EVT attempt (Fig. 4). However, there were no improvements in the functional outcomes of 3 patients who underwent a second EVT or in 2 patients who were monitored and followed up without further treatment. In the latter group, one patient had a worsened functional outcome at the last follow-up.

Among the 4 patients who had treatment-related complications, 3 had worsened outcomes: one had PICA territory infarction after repeated EVT for incomplete occlusion of cervical SDAVF, one had PICA territory infarction after repeated EVT for recurrent cervical SDAVF, and one had vertebral artery dissection after initial EVT for cervical SDAVF (Table 4).

### Treatment of recurrent SDAVFs after initial EVT

Of the 37 patients with complete occlusion by initial EVT, recurrence of SDAVF detected by spine MRI at the last follow-up was observed in 8 (21.6%), and follow-up spinal angiography was performed in 4 patients to determine additional treatment. Improved functional outcomes after complete occlusion were achieved in 2 of 3 patients who underwent surgery for recurring SDAVF. In contrast, in one patient who underwent repeated EVT for recurrent cervical SDAVF, complete occlusion was not achieved, and the patient had a worsened functional outcome. The remaining 4 patients who showed recurrence on spine MRI were still followed up without spinal angiography due to their stationary neurological state.

## Discussion

### Occlusion of SDAVF by initial EVT

In the present study, the rate of complete occlusion of the SDAVF by initial EVT using NBCA was 66.1%, which is similar to the results of previous retrospective studies and meta-analyses^4,10,17–19^. However, complete occlusion of the SDAVF was achieved in 53 of 56 patients (94.6%) at the last follow-up, including patients who underwent additional treatments such as surgery and repeated EVT.

We identified that the presence of multiple feeders was significantly associated with incomplete occlusion of the SDAVF by initial EVT (P < 0.001). The present study also showed that the rate of occlusion by initial EVT was significantly lower in patients with cervical SDAVFs than in those with SDAVFs in other locations (P = 0.04). The reason for the higher rate of incomplete occlusion of cervical SDAVF by initial EVT is thought to be due to the presence of multiple feeders from the PICA in addition to those from the deep cervical artery^20,21^.

### Treatment outcomes after initial EVT

In the present study, functional deterioration was prevented in 27 of 37 patients (73.3%) when complete occlusion of the SDAVF was achieved by an initial EVT attempt. Westphal et al. reported no differences in treatment outcomes after initial surgery, initial embolization, and a combined procedure when obliteration of SDAVFs was achieved^11^. In contrast, in the present study, among 19 patients in whom initial EVT did not achieve complete occlusion of the SDAVF, improved or stationary functional outcomes were achieved in 13 of 14 patients who underwent surgical treatment for residual SDAVF. Moreover, among 4 patients who needed additional treatment for recurrence, improved functional outcomes could be achieved only by surgery, whereas repeated EVT resulted in worsened outcomes without complete occlusion. Therefore, in cases of incomplete occlusion of the SDAVF after initial EVT, the present study showed that functional outcomes were dependent upon complete occlusion of the SDAVF by additional treatment and procedure-related complications.

Owing to the technical advancement in EVTs that has been made over the last decades^22^, the use of EVT as a first treatment attempt is expected to be more common in the treatment of SDAVF considering its advantages over spinal surgery. For instance, first EVT attempt may be a reasonable option in elderly patients and patients with obesity or other comorbidities who have higher risk of postoperative complications such as deep vein thrombosis or surgical site infection^23,24^. For the same reason, clinicians may favor the endovascular treatment in the case of incomplete occlusion by first EVT or recurrent case considering the advantages over the surgery. However, as shown in the present study, we recommend additional surgery as a salvage treatment rather than repeated EVT in the case of incomplete occlusion or recurrence.

Regarding the reasons why the likelihood of complete occlusion may be higher with surgery in the case of incomplete occlusion by initial EVT or of recanalization after initial complete occlusion, we speculate that collateral channels may develop and contribute AV shunt flow because the initial EVT blocks the main arterial feeders. These small multiple collaterals may not be accessible or occluded by further repeated EVT(s).

Therefore, we recommend surgery after incomplete occlusion by initial EVT rather than a second EVT procedure or close monitoring with the expectation of spontaneous thrombosis^7,25^.

Additionally, the present study found that the pretreatment duration of neurological symptoms was not significantly associated with a worsened outcome, contrary to a previous study that reported a higher rate of disability in SDAVF patients with delayed diagnoses^5^. The negative effect of symptom duration before treatment on long-term outcomes is still inconclusive, with conflicting results among studies^1,22^.

In previous studies, the severity of preoperative neurological deficits was a strong negative prognostic factor in symptomatic SDAVF^26,27^. However, it is remarkable that 73.3% of the patients with a pretreatment ALS score between 5 and 8 achieved improved functional outcomes in the present study. Additionally, pretreatment severity assessed by the ALS was not associated with a worsened outcome. Therefore, it would be unreasonable to consider preoperative severity or the severity of myelopathy in decision making regarding the aggressiveness of treatment, as suggested in clinical guidelines for the treatment of degenerative cervical myelopathy^28^. For instance, we could not justify the implementation of less-invasive treatments with a higher likelihood of incomplete occlusion of the SDAVF in patients with severe neurological symptoms due to myelopathy.

### Considerations in the treatment of cervical SDAVF

With the exception of the 4 reported procedure-related complications, all of which occurred during EVT of cervical SDAVF in the present study, there were no remarkable complications reported in patients with SDAVF at locations other than the cervical spine. A lower rate of complete occlusion by initial EVT in patients with cervical SDAVFs than in those with SDAVFs at other locations (33.3% vs. 72.3%, P = 0.04) was also observed in the present study. Although EVT is favored as an initial treatment option for cervical SDAVFs in the condition of subarachnoid hemorrhage, considering both the risk of complications and the incomplete occlusion rate of repeated EVT, additional surgical treatment should be considered when SDAVFs are incompletely occluded by initial EVT^29,30^.

### Limitations of the study

The present study was limited by its retrospective nature and the small number of patients included. Although this study identified a potential relationship between incomplete occlusion by initial EVT and multiple SDAVF feeders, outcome assessments of each subgroup that experienced incomplete occlusion by initial EVT are still inconclusive owing to the small sample size.

However, the present study was the first to analyze the treatment outcome of patients with SDAVF according to different treatment courses after a trial of initial EVT in both a statistical and descriptive manner. In a future study, it is necessary to investigate the outcome of SDAVF with incomplete occlusion after initial EVT by comparing a group who underwent additional surgical treatments with a group who underwent a second EVT in a prospective manner.

## Conclusion

In the treatment of SDAVFs, improved or stationary functional outcomes can be achieved after complete occlusion of the SDAVF by a trial of initial EVT at the same session of diagnostic spinal angiography. However, when deciding treatment options, the feasibility of EVT is the most important to achieve the best treatment outcome. Surgical treatment is still the first option when EVT is not feasible after diagnostic spinal angiography. Additionally, in cases of incomplete occlusion by initial EVT, surgical treatment should be considered rather than additional EVT or a “wait-and-see” approach with the expectation of spontaneous thrombosis to achieve complete obliteration of the SDAVF and improved functional outcomes. The presence of multiple feeders was significantly associated with incomplete occlusion by initial EVT. Considering the lower rate of complete occlusion and higher probability of procedure-related complications of repeated EVT for cervical SDAVF, surgery should be considered instead of repeated EVT when cervical SDAVF is incompletely occluded by initial EVT.

## Supplementary Information

Supplementary Information.

## Data Availability

The authors confirm that the data supporting the findings of this study are available within its supplementary materials.

## Code availability

The computing environment R Version 3.6.3 (R Development Core Team, 2017. R: A language and environment for statistical computing. R Foundation for Statistical Computing, Vienna, Austria. https://www.R-project.org/) was used for statistical analysis.
